# Dynamic reorganization of the AC16 cardiomyocyte transcriptome in response to TNFα signaling revealed by integrated genomic analyses

**DOI:** 10.1186/1471-2164-15-155

**Published:** 2014-02-24

**Authors:** Xin Luo, Minho Chae, Raga Krishnakumar, Charles G Danko, W Lee Kraus

**Affiliations:** 1 Laboratory of Signaling and Gene Regulation, Cecil H. and Ida Green Center for Reproductive Biology Sciences, University of Texas Southwestern Medical Center, Dallas, TX 75390, USA; 2 Division of Basic Research, Department of Obstetrics and Gynecology, University of Texas Southwestern Medical Center, Dallas, TX 75390, USA; 3 Graduate School of Biomedical Sciences, Program in Genetics and Development, University of Texas Southwestern Medical Center, Dallas, TX 75390, USA; 4 Department of Molecular Biology and Genetics, Cornell University, Ithaca, NY 14850, USA; 5 Graduate Field of Biochemistry, Molecular and Cell Biology, Cornell University, Ithaca, NY 14853, USA; 6 Department of Biological Statistics and Computational Biology, Cornell University, Ithaca, NY 14850, USA; 7 Current address: Institute for Regenerative Medicine, University of California, San Francisco 94143, USA

## Abstract

**Background:**

Defining cell type-specific transcriptomes in mammals can be challenging, especially for unannotated regions of the genome. We have developed an analytical pipeline called groHMM for annotating primary transcripts using global nuclear run-on sequencing (GRO-seq) data. Herein, we use this pipeline to characterize the transcriptome of an immortalized adult human ventricular cardiomyocyte cell line (AC16) in response to signaling by tumor necrosis factor alpha (TNFα), which is controlled in part by NF-κB, a key transcriptional regulator of inflammation. A unique aspect of this work is the use of the RNA polymerase II (Pol II) inhibitor α-amanitin, which we used to define a set of RNA polymerase I and III (Pol I and Pol III) transcripts.

**Results:**

Using groHMM, we identified ~30,000 coding and non-coding transcribed regions in AC16 cells, which includes a set of unique Pol I and Pol III primary transcripts. Many of these transcripts have not been annotated previously, including enhancer RNAs originating from NF-κB binding sites. In addition, we observed that AC16 cells rapidly and dynamically reorganize their transcriptomes in response to TNFα stimulation in an NF-κB-dependent manner, switching from a basal state to a proinflammatory state affecting a spectrum of cardiac-associated protein-coding and non-coding genes. Moreover, we observed distinct Pol II dynamics for up- and downregulated genes, with a rapid release of Pol II into productive elongation for TNFα-stimulated genes. As expected, the TNFα-induced changes in the AC16 transcriptome resulted in corresponding changes in cognate mRNA and protein levels in a similar manner, but with delayed kinetics.

**Conclusions:**

Our studies illustrate how computational genomics can be used to characterize the signal-regulated transcriptome in biologically relevant cell types, providing new information about how the human genome is organized, transcribed and regulated. In addition, they show how α-amanitin can be used to reveal the Pol I and Pol III transcriptome. Furthermore, they shed new light on the regulation of the cardiomyocyte transcriptome in response to a proinflammatory signal and help to clarify the link between inflammation and cardiomyocyte function at the transcriptional level.

## Background

The repertoire of coding and non-coding transcripts expressed in a given cell type - the “transcriptome” - reflects the specific biology of that cell type, including responses to external stimuli. Thus, information about the transcriptome can provide deep biological insights with relevance to physiology and disease. Determining and analyzing the complete transcriptome, however, can be challenging, especially with respect to unannotated cell type-specific transcripts. This endeavor, however, has been facilitated by computational genomics approaches that leverage deep sequencing technologies. Herein, we apply these approaches to the study of the cardiomyocyte transcriptome, which has revealed interesting new information related to cardiovascular disease (CVD).

CVD is the leading cause of death worldwide [1]. Many of the underlying pathologies of CVD are directly or indirectly associated with inflammation. Many studies have focused on the effects of inflammation on endothelial function and atherosclerosis [2-4]. However, the detrimental effects of inflammation are not limited to the vascular system, but also occur in cardiomyocytes. The progression from heart injury to heart failure is closely linked to necrosis, apoptosis, or autophagy in cardiomyocytes [5,6]. During heart failure, cardiomyocytes serve as the major source of cytokine secretion, and the secreted cytokines not only interfere with the function of the cardiomyocytes, but also recruit cardiac fibroblast cells, causing fibrosis and eventually heart damage and infarction [7,8].

Although the effects of inflammation in cardiomyocytes have been examined previously [9,10], the detailed mechanisms underlying these effects are poorly understood. NF-κB, a key transcriptional regulator of inflammation, has been shown to play a dual role in CVD through its actions in various cell types of the cardiovascular system. It promotes an anti-apoptotic cardioprotective effect during hypoxia and reperfusion injury by repressing genes involved in cell death pathways, but also supports the secretion of detrimental cytokines during acute or chronic inflammatory injury, leading to cell death and fibrosis [11,12]. The specific regulatory effects of NF-κB on gene expression programs in cardiomyocytes are not well understood.

Cellular functions and processes are largely determined by carefully orchestrated cell type-specific gene-expression programs. For example, a recent study has characterized an extensive estrogen-regulated gene expression program in breast cancer cells that alters a large fraction of the transcriptome and promotes a mitogenic growth program [13,14]. A greater understanding of the NF-κB-dependent proinflammatory gene expression program in cardiomyocytes will provide a greater understanding of the links between inflammation and impaired cardiomyocyte function. Non-coding RNAs (ncRNAs) should be a key component of this analysis since previous studies have demonstrated key roles for ncRNAs, including microRNAs (miRNAs) and long non-coding RNAs (lncRNAs), in cardiovascular function [15,16]. Further mapping and characterization of all functional transcripts, including those generated by RNA polymerases I and III, are necessary for a complete picture of the cardiomyocyte transcriptome.

In the studies described herein, we have used a combination of genomic approaches, including GRO-seq and ChIP-seq, to characterize the transcriptome of AC16 immortalized adult human ventricular cardiomyocyte cells in response to tumor necrosis factor (TNFα). Our studies shed new light on the regulation of the cardiomyocyte transcriptome in response to a proinflammatory signal and help to clarify the link between inflammation and cardiomyocyte function at the transcriptional level.

## Results

### AC16 cells respond to TNFα stimulation by activating an NF-κB-dependent signaling pathway

**Figure 1:**
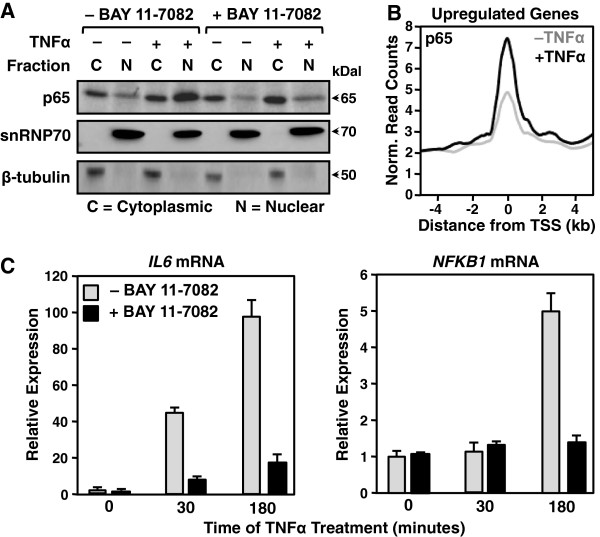
**TNFα stimulation of AC16 cells activates the NF-κB signaling pathway. A)** Western blot of the NF-κB p65 subunit, snRNP70 (a nuclear marker), and β-tubulin (a cytoplasmic marker) using cytoplasmic and nuclear fractions from control and TNFα-treated AC16 cells (25 ng/ml for 30 min.) with and without the IKKα/β inhibitor BAY11-7082 (5 μM pretreatment for 1 hour). **B)** Metagene representation showing the average ChIP-seq read density of the NF-κB p65 subunit as a function of distance from the TSSs (± 4 kb) of upregulated protein-coding genes (defined by GRO-seq). The line shading indicates the control (*grey*) and TNFα-treated (*black*) conditions. **C)** RT-qPCR analysis of *IL6* (*left*) and *NFKB1* (*right*) mRNA expression in control and TNFα-treated AC16 cells (25 ng/ml TNFα for 30 or 180 minutes). The bar colors indicate the control (*grey*) and BAY11-7082-treated (*black*) samples. Each data point represents the mean + SEM for three independent biological replicates.

To investigate the molecular aspects of proinflammatory gene regulation in AC16 cells, we first characterized responses triggered by stimulation with tumor necrosis factor alpha (TNFα). We expected TNFα to activate the NF-κB signaling pathway, as has been reported previously for macrophages and endothelial cells [17]. To verify this, we monitored NF-κB activation in AC16 cells following TNFα stimulation by Western blotting fractionated cell extracts. TNFα induced NF-κB nuclear translocation, which was blocked by the IKKα/β inhibitor BAY 11–7082 (Figure 1A). TNFα also promoted the recruitment of NF-κB to chromatin globally, as assayed by ChIP-seq for the NF-κB p65 subunit (Figure 1B). Finally, TNFα simulated the expression of known NF-κB-dependent proinflammatory genes, such as *IL6* and *NFKB1* in a manner that was substantially reduced by BAY11-7082 (Figure 1C). Taken together, these results demonstrate that NF-κB is essential for the activation of a set of proinflammatory target genes in TNFα-treated AC16 cells.

### The proinflammatory AC16 transcriptome includes a diverse array of coding and non-coding transcripts

**Figure 2:**
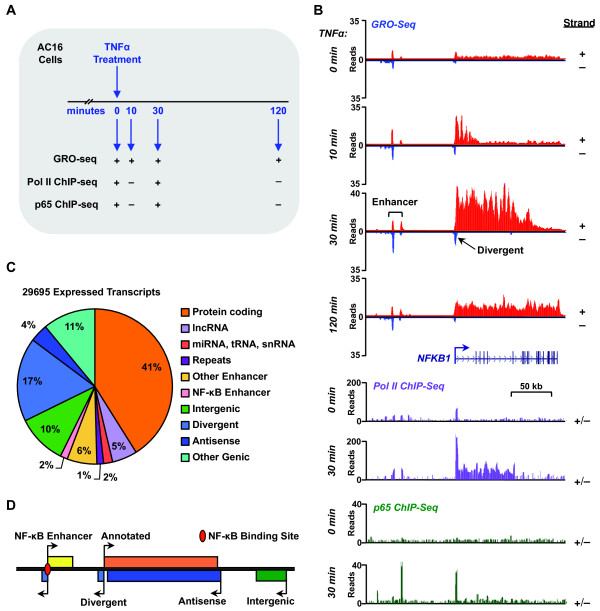
**Defining the AC16 transcriptome using GRO-Seq. A)** Overview of the experimental scheme and treatments for the GRO-seq and ChIP-seq experiments in AC16 cells. **B)** Genome-browser view of the genomic region around the *NFKB1* gene showing the distribution of GRO-seq reads, and Pol II and NF-κB p65 ChIP-seq reads in control and TNFα-treated AC16 cells at the indicated time points. **C)** Classification of all expressed transcripts in AC16 cells. Pie chart showing the composition of the AC16 transcriptome based on known and de novo annotations and functional assignments. **D)** Schematic representation of some of the transcript types listed in panel **(C)**.

To better understand the AC16 transcriptome, we used global run-on coupled with deep sequencing (GRO-seq), a direct, high throughput genomic method, which maps the position and orientation of all transcriptionally engaged RNA polymerases across the genome with high spatial resolution [18]. As such, GRO-seq provides a sensitive map of all regions in the genome actively transcribed by RNA polymerases I, II, and III (Pols I, II, and III) [14]. We performed GRO-seq after a short time course of TNFα treatment (0, 10, 30, and 120 min.; Figure 2A). When visualized using a genome browser, the data reveal a sensitive and accurate strand-specific approach for capturing the immediate transcriptional effects of TNFα that is more sensitive than Pol II ChIP-seq (Figure 2B). For example, for the classic inflammatory transcription factor gene *NFKB1*, GRO-seq reveals the time-dependent progression of Pol II waves moving from the 5′ to 3′ end of the transcription unit during the TNFα-treatment time course (Figure 2B), information that can be used to determine rates of transcription (~3 kb per minute in the case of *NFKB1*; [19]). Moreover, GRO-seq also reveals the expression of a divergent transcript generated from the *NFKB1* promoter, as well as bi-directional enhancer transcripts (eRNAs) originating ~50 kb upstream of the *NFKB1* promoter, which may mark functional enhancers for *NFKB1* (Figure 2B).

To identify all transcripts in the proinflammatory AC16 transcriptome, including previously unannotated transcripts, we combined GRO-seq with a bioinformatics approach called groHMM, which uses a two-state hidden Markov model to identify active transcription units genome-wide [13]. Using this approach, we identified 29,695 transcripts that are expressed in AC16 cells during at least one time point during the course of TNFα treatment (see Methods for details). To ascertain the potential functional role of each transcript, we compared the genomic locations of the identified transcription units with existing genomic annotations. We found that approximately half of the transcription units discovered in our GRO-seq data can be mapped to annotated regions, including genes encoding proteins, long non-coding RNAs (lncRNAs), microRNAs (miRNAs), tRNAs, snRNAs, and repeat elements (Figure 2C), many of which are relevant to cardiac biology (e.g., the mRNA *CFLAR*, the lncRNA *MALAT1*, the microRNA 21 precursor *MIR21*; Additional file 1). The remaining transcription units map to genomic loci that were previously unannotated, but may harbor important genetic information and support important functions within the TNFα response in cardiomyocytes (Figure 2C). We categorized these unannotated transcription units based on their orientation and location relative to annotated genes, including divergent, antisense, and intergenic (Figure 2D). The intergenic transcripts include a category of short, bidirectionally transcribed eRNAs, as we have described previously [20].

### AC16 cells rapidly and dynamically reorganize their transcriptomes in response to TNFα

**Figure 3:**
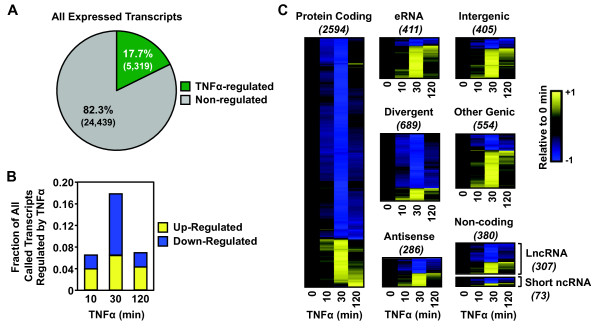
**Global reorganization of the AC16 cell transcriptome in response to TNFα.** GRO-seq in AC16 cells treated with a time course of TNFα reveals dramatic effect of signaling on the transcriptome. **A)** Pie chart showing the fraction of all transcripts expressed in AC16 cells that are regulated by TNFα at any treatment time point. **B)** Bar graph showing the fraction of all transcripts expressed in AC16 cells that are up- or downregulated in response to TNFα at the indicated time points. **C)** Heatmap representations of TNFα regulation of different classes of transcripts at the indicated time points. The transcripts in each class were clustered by their time-dependent expression levels using a hierarchical clustering algorithm. The color-based scale represents GRO-seq reads at the indicated time points scaled to the read density at time zero. In this representation, the x-axis (columns) is time of TNFα treatment and the y-axis (rows) is the individual transcripts. From top to bottom, the information has no particular order, although the clustering algorithm separates the distinct patterns of regulation. For example, those that are down regulated (blue) are at the top and those that are upregulated (yellow) are at the bottom.

To investigate the effects of TNFα on the AC16 transcriptome, we analyzed changes caused by TNFα treatment in further detail. We used edgeR, a program that determines differential expression of replicated count data considering biological and technical variability [21], to identify transcription units whose expression changes during the time course of TNFα treatment. This analysis revealed that a large fraction (~18%) of expressed transcripts is regulated in response to TNFα in a surprisingly rapid manner (Figure 3A and B). The onset of regulation is evident as early as 10 minutes after treatment in many cases (~6% of transcripts; Figure 3B), similar to what we have observed previously following estrogen stimulation in MCF-7 cells [13]. By 30 minutes, the majority of regulated transcripts have reached their maximal change, with most reflecting decreased expression (Figure 3B and C). Interestingly, the majority of genes (both up- and down-regulated) returned to near-homeostatic levels 120 minutes post-treatment (Figure 3C). This temporal pattern follows a similar time scale as the oscillating patterns of activation and nuclear localization of NF-κB previously observed [22]. These changes are most likely explained as a direct readout of NF-κB’s presence on chromatin.

When analyzing the response of individual classes of transcripts, we found that small non-coding RNAs, lncRNAs, divergent RNAs, and antisense RNAs are up- and down-regulated with similar ratios and kinetics as protein-coding transcripts (Figure 3C). Conversely, intergenic and enhancer transcripts are enriched for upregulation (p < 4 × 10^-25^; Fisher’s exact test) at every time point of TNFα treatment, which is consistent with their putative gene activation function (Figure 3C). Overall, these analyses reveal a dynamic regulation of the AC16 transcriptome by TNFα that fits with the logic of a proinflammatory stress response: broad repression of transcription, with rapid and robust activation of a selected set of target genes. This pattern of regulation is distinct from the mitogenic transcriptional response that we have characterized previously [13,14].

### GRO-seq reveals different dynamics for the TNFα-dependent activation and repression of transcription

**Figure 4:**
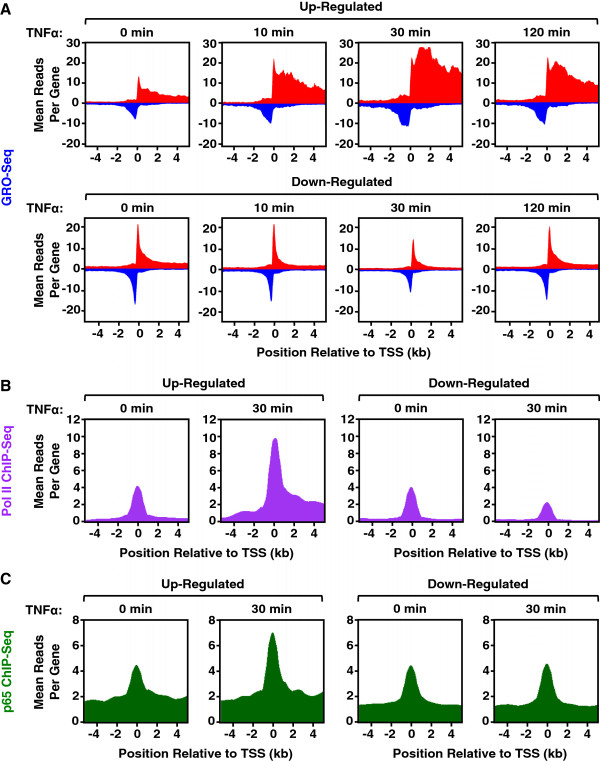
**GRO-seq and Pol II ChIP-seq reveal the dynamics of TNFα-dependent transcription in AC16 cells.** GRO-seq and ChIP-seq in AC16 cells treated with a time course of TNFα reveals the dynamics of transcription. **A)** Metagene representations showing the average GRO-seq read distributions ± 4 kb around the TSSs of upregulated (*top*) or downregulated (*bottom*) RefSeq genes over a time course of TNFα treatment in AC16 cells. **B** and **C)** Metagene representations showing the average Pol II **(B)** and NF-κB p65 **(C)** ChIP-seq read distributions ± 4 kb around the TSSs of upregulated (*left*) or downregulated (*right*) RefSeq genes at 0 and 30 min of TNFα treatment.

GRO-seq affords the opportunity to examine the dynamics of transcription on a short time scale. To examine the dynamics of Pol II in response to TNFα, we focused on the time-dependent redistribution of Pol II at upregulated and downregulated RefSeq genes. Metagene analyses showing the average GRO-seq signal mapped to ± 4 kb around the transcription start sites (TSSs) of all genes of interest reveal distinct Pol II dynamics for upregulated and downregulated genes (Figure 4A). For genes upregulated upon TNFα treatment, Pol II rapidly increased and released into the gene body, with a limited time spent at promoter-proximal pause sites, which is consistent with previously characterized effects of the TNFα/NF-κB signaling pathway on transcriptional elongation [19]. The activation occurred as early as 10 min., was maximal at 30 min., and was partially attenuated by 120 min. (Figure 4A, top row). In contrast, for genes downregulated upon TNFα treatment, an accumulation of promoter-proximally paused Pol II was evident prior to TNFα treatment and was only reduced after 30 min. of TNFα treatment. A reduction in gene body Pol II, however, was evident as early as 10 min. following TNFα treatment. The levels of promoter-proximally paused Pol II and gene body Pol II returned to basal levels after 120 min. (Figure 4A, bottom row). Interestingly, Pol II shows different dynamics during an acute TNFα-dependent transcriptional response in AC16 cells than it does during a rapid estrogen-dependent mitogenic response in MCF-7 breast cancer cells. Specifically, estrogen upregulated genes show a greater induction of promoter-proximally paused Pol II in response to the estrogen stimulus, suggesting a greater effect on Pol II loading or initiation than elongation [13,19]. These results highlight the distinct gene activation mechanisms mediated by NF-κB and ERα. Pol II ChIP-seq shows a pattern of Pol II dynamics consistent with those observed by GRO-seq for both up- and downregulated genes (Figure 4B). In addition, ChIP-seq shows the induced binding of NF-κB at the promoters of upregulated genes, but not downregulated genes, suggesting NF-κB dependence for TNFα-mediated upregulation, but not downregulation (Figure 4C).

### α-Amanitin identifies Pol I, Pol II, and Pol III activity across the AC16 transcriptome

Three different RNA polymerases produce the mammalian cell transcriptome: Pol I transcribes a large transcript from each of the ribosomal DNA (rDNA) loci, which is later cleaved into 18 s, 5.8 s, and 28 s rRNAs, accounting for 50% of the total synthesized RNAs in the cell [23]; Pol II synthesizes the precursor RNAs for mRNAs and most lncRNAs, microRNAs and snRNAs; and Pol III transcribes the 5 s rRNAs, tRNAs, and other small RNAs closely associated with housekeeping functions [24]. Although the regulation and function of Pol II has been well studied, and recent mapping of the localization of the Pol III transcription machinery genome-wide has shed some light on its transcription profile [25-27], many questions remain regarding the coordination of Pol I, II, and III activities. For example, which polymerase controls synthesis of novel unannotated transcripts? How are Pol II- and non-Pol II-transcribed regions distributed across the genome?

**Figure 5:**
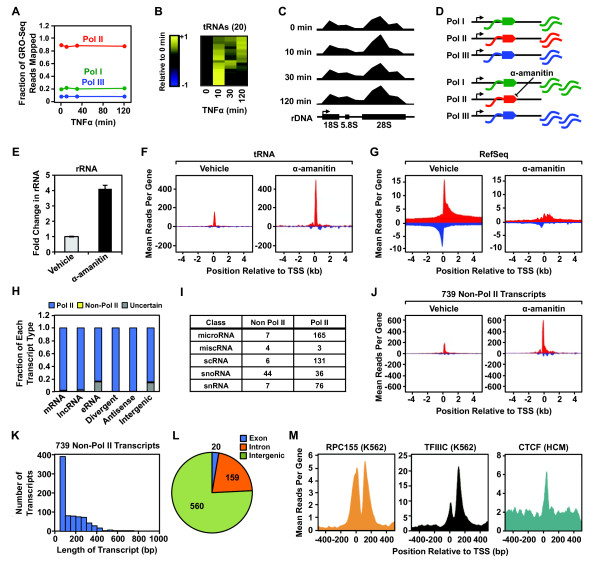
**Pol I, II, and III transcription in AC16 cells in response to TNFα.** AC16 cell nuclei were treated with α-amanitin for 15 min. prior to GRO-seq analysis. The final read density for each gene was normalized to the total reads obtained in each condition. **A)** Effect of TNFα on the fraction of GRO-seq reads mapped to rDNA repeats (Pol I; *green line*), RefSeq genes (Pol II; *red line*), and tRNA genes (Pol III; *blue line*) over the time course of TNFα treatment. **B)** Heatmap showing the expression of 20 tRNA transcripts upregulated during a time course of TNFα treatment. **C)** Browser track representation of GRO-seq reads mapped to rDNA repeats (GenBank U13369.1) in 1 kb bins over a time course of TNFα treatment. **D)** Scheme for the GRO-seq experiments with α-amanitin showing the expected effects on Pol I, II, and III transcription. **E)** Relative change in GRO-seq reads at rDNA repeats in control and α-amanitin-treated AC16 nuclei. **F** and **G)** Metagene representations of the average number of GRO-seq reads distributed around tRNA **(F)** and RefSeq **(G)** gene TSSs in control and α-amanitin-treated AC16 nuclei. **H)** Fraction of different types of uniquely mapped transcripts transcribed by Pol II or other RNA polymerases, as revealed by α-amanitin treatment. **I)** The number of annotated, short, non-coding transcripts transcribed by Pol II or other RNA polymerases, as revealed by α-amanitin treatment. **J)** Metagene representations of the average GRO-seq read distributions ± 4 kb around the TSSs of 739 non-Pol II transcripts identified using α-amanitin. **K)** Histogram showing the length distribution of all 739 non-Pol II transcripts from **(J)**. **L)** Pie chart showing the genomic distribution of the genes encoding all 739 non-Pol II transcripts from **(J)**. **M)** Metagene representations of the average ChIP-seq read distributions ± 500 bp around the TSSs for RPC155 (Pol III subunit) in K652 cells (*left*), TFIIIC in K562 cells (*middle*), and CTCF in human cardiomyocytes (HCM) (*right*) relative to the TSSs (± 500 bp) of the identified non-Pol II transcripts.

With appropriate mapping techniques, GRO-seq allows the detection of RNA polymerases density on tRNA genes and rRNA genes [13]. Whereas tRNA genes have enough sequence variation to allow unique mapping of GRO-seq reads, rRNA genes do not. Thus, we created a single reference rRNA gene to which all rRNA reads are mapped, yielding an average response across all rRNA genes [13]. Although transcription of 20 tRNA genes is rapidly and transiently upregulated in response to TNFα (Figure 5A and B), transcription of the remaining tRNA and rRNA genes, on average, is largely unaffected (Figure 5A and C).

To obtain a greater understanding of the AC16 transcriptome and to investigate coordination among the different RNA polymerases in TNFα-induced inflammatory responses in cardiomyocytes, we used α-amanitin to distinguish between the activities of Pol II (sensitive to the concentration of α-amanitin used) and Pol I/III (not sensitive). Nuclei isolated from AC16 cells were incubated on ice with α-amanitin for 15 min. prior to the run-on reaction that generates the short bromouridine-labeled transcripts for detection by GRO-seq. Since the final read density of each gene is normalized to the total reads obtained in each condition, non-Pol II transcripts are relatively enriched due to the loss or reduction of Pol II transcripts in the α-amanitin-treated condition (Figure 5D). As expected, we observed a relative enrichment of GRO-seq signals from rDNA repeats (Pol I) and tRNA genes (Pol III), as well as a reduction of the GRO-seq signal from annotated RefSeq genes (mostly Pol II) (Figure 5E, F, and G). This pattern serves as a validation of the reliability of our approach in mapping Pol II and non-Pol II transcripts.

Next, we compared the GRO-seq reads at uniquely mapped transcripts between α-amanitin- and vehicle-treated nuclei to determine which types of transcripts were produced by Pol II or Pol I/III (“non-Pol II transcripts”). These results indicate that most of the recently defined types of long non-coding transcripts, such as lncRNAs, eRNAs, divergent RNAs, and antisense RNAs are transcribed by Pol II (Figure 5H; Additional file 2), whereas annotated short non-coding transcripts are distributed between the Pol II and non-Pol II categories (Figure 5I). For example, the majority of small nucleolar RNAs (snoRNAs) and small cytoplasmic RNAs (scRNAs) are transcribed by Pol II, whereas small nuclear RNAs (snRNAs) are transcribed by both Pol II and Pol III, as expected (Figure 5I).

### Characterization of the Pol I/III transcriptome in AC16 cells

To further characterize the non-Pol II transcriptome in AC16 cells, we mapped 739 non-Pol II transcripts from GRO-seq data generated ± α-amanitin (Figure 5J). We assume that most are transcribed by Pol III, since Pol I mainly controls transcription from the rDNA repeats, although we did not confirm this experimentally. This set of non-Pol II transcripts includes mainly tRNAs, rRNAs, some snRNAs, and transcripts generated from SINE repeat elements, as well as 172 novel, previously unannotated transcripts (Additional file 3). The lengths of the majority of the 739 primary non-Pol II transcripts are <400 nucleotides, which indicates that they are short, non-coding RNAs (Figure 5K). These transcripts originate mostly from intergenic regions and, to a lesser extent, intronic regions. Only a few transcripts were mapped to the exons of genic regions, concentrated in the 5′ or 3′ UTRs (Figure 5L; Additional file 3).

As expected, a large fraction of the non-Pol II transcripts that we identified overlap with the Pol III machinery (49% and 39% respectively), as indicated by ChIP-seq of the Pol III subunit RPC155 or the Pol III transcription factor TFIIIC (ENCODE data from K562 cells) (Figure 5M), further verifying our ability to identify Pol III transcripts. Many of the transcripts also overlap with CTCF binding sites (33%), which suggests an insulator-like function related to the genes encoding these transcripts. Interestingly, with exception of the aforementioned upregulated tRNA genes (Figure 5B), the expression pattern of the rest of the non-Pol II transcripts remained fairly constant across the time course of TNFα treatment [data not shown; edgeR failed to identify statistically significant regulated genes at any time point of TNFα treatment with a false discovery rate (FDR)-corrected q value threshold (q < 0.001)]. Thus, during the TNFα-induced inflammatory response, transcriptional regulation occurs mostly for Pol II transcripts, but not to a great extent for Pol I and Pol III transcripts.

### GRO-seq identifies enhancers in TNFα-stimulated cardiomyocytes

**Figure 6:**
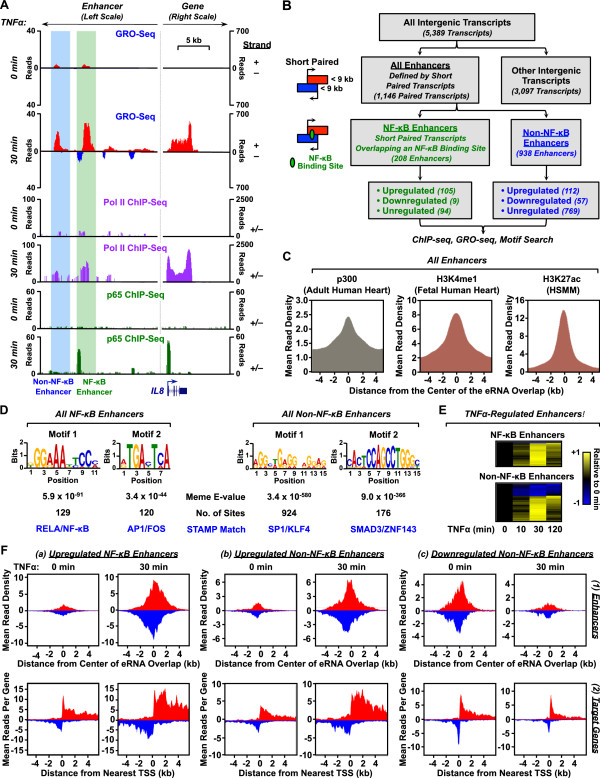
**Enhancer transcripts in AC16 cells originate from NF-κB-dependent and NF-κB-independent genomic loci. A)** Genome browser tracks showing read distributions for GRO-seq, Pol II ChIP-seq, and p65 ChIP-seq at the promoter and distal enhancers of the *IL8* gene. The blue-shaded genomic region shows an NF-κB-independent enhancer, whereas the green-shaded genomic region shows a NF-κB-dependent enhancer. A schematic of the *IL8* gene annotation is shown and the length scale is indicated. **B)** Flowchart of enhancer classification in AC16 cells based on genomic location, eRNA production, length of the transcribed regions, overlap with NF-κB binding, and TNFα-mediated regulation. **C)** Metagene representations of the average ChIP-seq read distributions for p300 in adult human heart (*left*), H3K4me in fetal human heart (*middle*), and H3K27ac in human skeletal muscle myoblasts (HSMM) (*right*) for all 1,146 enhancers identified by GRO-seq, shown relative to the midpoint of overlap of the bidirectionally transcribed eRNAs (± 4 kb). **D)** De novo motif analyses of 208 NF-κB-dependent enhancers (*left*) and 938 NF-κB-independent enhancers (*right*) using MEME/STAMP. The top two most enriched motifs for each category are shown. **E)** Heatmap representations of TNFα regulation of enhancer transcription for NF-κB-dependent (*top*) and NF-κB-independent (*bottom*) enhancers. The color-based scale represents GRO-seq reads at the indicated time points scaled to the read density at time zero. **F)** Metagene representations of the average GRO-seq read distributions ± 4 kb around (1) the midpoint of overlap of the bidirectionally transcribed eRNAs (*top row*) or (2) the TSSs of the nearest neighboring protein-coding or lncRNA putative target genes (*bottom row*) for the following groups of enhancers: **(a)** TNFα-upregulated NF-κB-dependent enhancers (*left*), **(b)** TNFα-upregulated NF-κB-independent enhancers (*middle*), and **(c)** TNFα-downregulated NF-κB-independent enhancers (*right*).

Recent studies have shown that transcription factor binding sites are focal points for the recruitment of Pol II and the production of characteristic short, mono- or bi-directional transcripts called enhancer RNAs (eRNAs) [13,20,28-31]. For example, at the proinflammatory gene *IL8*, we observed two sites of TNFα-induced eRNA production about 15–20 kb upstream of the TSS (Figure 6A). Both sites showed TNFα-dependent accumulation of GRO-seq and Pol II ChIP-seq signals, while only the more proximal of the two sites was bound by NF-κB (Figure 6A). These enhancers and their associated eRNAs may play an essential role in TNFα-dependent activation of the nearby *IL8* gene.

We have recently developed a computational approach for identifying functional enhancers based on these patterns of transcription in GRO-seq data [20]. Using this approach, we identified 1,146 sites of paired intergenic eRNA production in AC16 cells (Figure 6B). Metagene analyses of ChIP-seq data from adult human heart, fetal human heart, and human skeletal muscle myotubes (HSMM) for the 1,146 putative enhancers showed expected patterns of enrichment for well characterized enhancer features, such as p300, H3K4me1, and H3K27ac (Figure 6C). Remarkably, the putative enhancers identified in AC16 cells by GRO-seq match well with enhancer features in the ChIP-seq data from related, but distinct, cell types.

MEME-based motif analyses [32,33] of the putative NF-κB and non-NF-κB enhancers defined by GRO-seq revealed enrichment of different DNA sequences, which were assigned to specific transcription factors using STAMP [34]. The NF-κB enhancers were highly enriched for the RELA/NF-κB motifs (Figure 6D, left panel) and NF-κB p65 binding (Additional file 4), as expected, as well as AP-1 and FOS motifs (Figure 6D, left panel). The latter is consistent with previous demonstrations that AP-1 augments the NF-κB regulatory program [35]. Interestingly, both NF-κB and AP-1 are activated during heart failure [36]. The non-NF-κB enhancers were enriched in motifs for the transcription factors Sp1, Krüppel-like factor 4 (KLF4), SMAD3, and ZNF143 (Figure 6D, right panel). Other motifs are consistent with previous literature as well. For example, Sp1 has consistently been found in searches for cardiac transcription factors and is associated with the regulation of many cardiac genes [37-39], KLF4 is a critical transcriptional regulator of stress responses in cardiomyocytes [25-27], Smad3 is a key mediator of cardiac inflammation and fibrosis [40], and ZNF143 is critical for heart development in zebrafish [41].

Signal-regulated expression of eRNAs is a common theme [13,20,29,30], an effect that we observed with the AC16 enhancers (Figure 6E). Specifically, our analyses revealed that 114 out of 208 (~55%) NF-κB binding site eRNAs are regulated by TNFα, with almost all upregulated, whereas only 169 out of 938 (~18%) non-NF-κB binding site eRNAs are regulated by TNFα, with two-thirds upregulated (Figure 6B, E, and F). Thus, the non-NF-κB binding site enhancers may represent a class of constitutive enhancers that control housekeeping functions in AC16 cells.

To further investigate the potential gene regulatory functions of the predicted NF-κB and non-NF-κB enhancers in AC16 cells, we assayed transcription levels by GRO-seq at the enhancers and their nearest annotated neighboring putative target genes with and without TNFα treatment (Figure 6F). We analyzed separately (1) upregulated NF-κB enhancers (left), (2) upregulated non-NF-κB enhancers (middle), and (3) downregulated non-NF-κB enhancers (right). Interestingly, transcription of the enhancers and target genes were well correlated (i.e., upregulation of enhancer transcription was correlated with an upregulation of target gene transcription, whereas downregulation of enhancer transcription was correlated with a downregulation of target gene transcription) (Figure 6F). These results provide further support for the functionality of the NF-κB and non-NF-κB enhancers predicted by GRO-seq.

### The TNFα-induced transcriptional response in AC16 cells reveals a functional link between inflammation and the biology of cardiomyocytes

**Figure 7:**
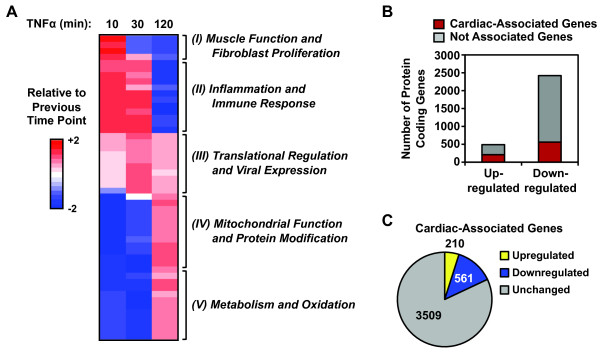
**The TNFα-induced transcriptional response in AC16 cells reveals a functional link between inflammation and cardiomyocyte biology. A)** The heatmap represents time-dependent changes in the enrichment of Gene Ontology (GO) terms in response to TNFα treatment. In this representation, the x-axis (columns) is time of TNFα treatment and the y-axis (rows) is the GO terms, with those that are enriched shown in red and those that are de-enriched shown in blue. The GO terms represent the properties of gene sets clustered based on related molecular functions and biological processes. We used Gene Set Enrichment Analysis (GSEA) to identify the top 25 enriched GO terms based on the TNFα significantly upregulated gene set and the top 25 de-enriched GO terms based on the TNFα significantly downregulated gene set. We then calculated the normalized enrichment score for each of the 50 GO terms at the indicated time points and clustered them based on the time-dependent changes using hierarchical clustering. Each line across represents the relative enrichment score of an enriched (in red) or de-enriched (in blue) GO term relative to the previous time point. The GO terms in each cluster are listed in Additional file 6. The number of GO terms in each cluster is: (I) 5, (II) 11, (III) 10, (IV) 12, and (V) 12. **B)** Many TNFα-regulated genes have cardiac-associated functions, as assigned by the Cardiovascular Gene Ontology Annotation Initiative. The bar graph shows the number of protein coding genes from the AC16 cell TNFα-regulated gene set that have or do not have cardiac-associated functions. **C)** Many of the 4,278 cardiac-associated genes identified by the Cardiovascular Gene Ontology Annotation Initiative project are regulated by TNFα. Pie chart showing the number of genes from the 4,278 cardiac-associated genes that are up-, down-, or unregulated by TNFα.

To relate the transcriptome changes to biological processes, we performed gene set enrichment analyses and gene ontology analyses on both TNFα up- and downregulated protein-coding genes identified by GRO-seq in the AC16 cell transcriptome (Figure 7A; Additional file 5). The biological functions associated with the up- and down-regulated gene sets are closely related to cardiac function. For example: (1) motor protein and myosin-related muscle functions are directly related to the electrophysiology of heart muscle [42], (2) fibroblast proliferation and endothelial-to-mesenchymal transition contribute to cardiac fibrosis [7,43]; and (3) mitochondrial function and lipid oxidation are closely related to normal cardiac physiology [44-46]. These dynamic transcriptome changes reflect the time-dependent shifting of biological processes in cardiomyocytes in response to TNFα (Figure 7A). Interestingly, genes related to muscle function and inflammation are upregulated immediately, whereas genes related to mitochondrial function and metabolism are downregulated first and upregulated later in the time course (Figure 7A). These results highlight the sequential transcriptional responses that underlie shifting cellular responses in cardiomyocytes in response to TNFα treatment.

We augmented this analysis using a database from the Cardiovascular Gene Ontology Annotation Initiative project, which contains more than 4,278 genes critical for cardiac physiology and pathology. A large fraction of both up- and downregulated genes are in the cardiac-associated gene list (Figure 7B) and ~20% are regulated by TNFα treatment (Figure 7C). Interestingly, 166 of the 1,146 enhancers predicted by GRO-seq are located near genes critical for cardiac physiology (data not shown). Collectively, our analyses of the TNFα-altered transcriptome indicate that the AC16 cellular state switches from maintenance of basal housekeeping functions to defense against inflammatory stress.

### TNFα-induced transcriptome changes result in corresponding alterations in the steady-state levels of mRNAs and proteins

**Figure 8:**
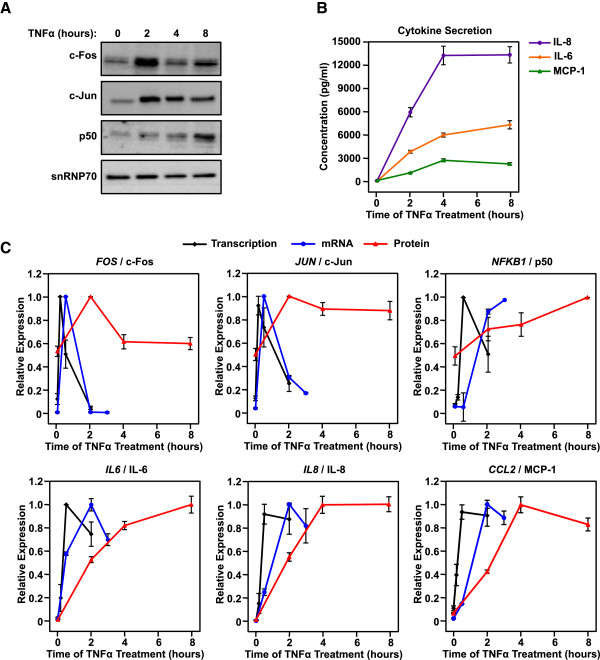
**TNFα induced transcriptional responses in AC16 cardiomyocytes result in corresponding changes in mRNA and protein levels with similar, but delayed, kinetics. A)** Western blots of c-Fos, c-Jun, the NF-κB p50 subunit, and snRNP70 (a loading control) from whole cell extracts of control and TNFα-treated AC16 cells (25 ng/ml of TNFα for the indicated treatment times). The assays were repeated three times. **B)** Scatter plots showing the concentration of secreted cytokines determined using a Bio-rad Bio-Plex cytokine assay, including IL-6, IL-8*,* and MCP-1 as indicated in control and TNFα-treated AC16 cells (25 ng/ml of TNFα for the indicated treatment times). Each data point represents the mean ± SEM for three independent biological replicates. **C)** Scatter plots showing the level of transcription (by GRO-seq), mature mRNA (by RT-qPCR), and protein (by Western blotting or Bio-Plex cytokine assay) for *FOS*, *JUN, NFKB1, IL6*, *IL8,* and *CCL2*, as indicated, in control and TNFα-treated AC16 cells (25 ng/ml of TNFα for the indicated treatment times). Each data point represents the mean ± SEM for two (GRO-seq) or three (RT-qPCR, Western, Bio-Plex cytokine assay) independent biological replicates.

As expected, the TNFα-induced changes in the AC16 transcriptome result in corresponding changes in mature mRNA and protein levels in a similar manner, but with delayed kinetics (Figure 8). For example, the robust upregulated transcription of key TNFα target genes (e.g., *FOS, JUN, NFKB1, IL6, IL8,* and *CCL2*) is followed by corresponding changes in the steady-state levels of the cognate mRNAs and proteins, with a delay of approximately 20 to 100 minutes for mRNA and 120 to 240 minutes for proteins. These results clearly illustrate how the dynamically regulated transcriptome alters the cellular proteome. These results also further support our observation that AC16 cardiomyocytes secrete cytokines in response to TNFα stimulation (Figure 8C). These cytokines may play an essential role in the overall effects of inflammation in cardiac biology.

### Role of non-coding RNAs and the TNFα-induced proinflammatory transcriptome

Protein-coding genes represent only part of the AC16 transcriptome; the functions carried out by the non-coding transcripts that we identified may also play critical roles in the inflammatory response in cardiomyocytes. Discerning the potential functions of ncRNAs can be difficult due to limited annotations and direct information available. To overcome these limitations, we performed gene ontology analyses using the Genomic Regions Enrichment of Annotations Tool (GREAT), which aids in predicting the molecular functions, associated biological processes, and disease associations based on the genomic region of interest and nearby genomic regions [47]. As such, GREAT has proven to be a powerful tool for studying cis-regulatory elements. Using GREAT, we found that TNFα-induced lncRNAs, eRNAs, and antisense transcripts are enriched in the same biological processes as the TNFα-induced protein-coding genes (Additional file 6). For example, both *CASP8 and FADD-like apoptosis regulator* (*CFLAR*) and its antisense transcript are upregulated upon TNFα stimulation (Additional file 1A). *CFLAR* is a crucial component of the signaling pathway involved in cardiac remodeling and heart failure [48]. In addition, the lncRNA *metastasis-associated lung adenocarcinoma transcript 1* (*MALAT1*), which is predicated to be downregulated in many types of heart disease by the NextBio-Disease Atlas (http://www.nextbio.com/b/search/da.nb), is downregulated upon TNFα stimulation (Additional file 1B). In addition to these antisense and lncRNAs, several primary microRNA transcripts that are associated with cardiac function and deregulated in pathological conditions of the cardiovascular system are regulated by TNFα. For example, *microRNA-21* (*miR-21*), an abundant microRNA whose primary transcript is upregulated by TNFα (Additional file 1C), is upregulated in many types of heart disease and may be a useful therapeutic target [49,50]. Moreover, the non-coding RNA *MIRLET7BHG*, which is a precursor for five microRNAs including let-7a3 and let-7b, is downregulated upon TNFα treatment (Additional file 1D). Let-7 family members are highly expressed in the heart and are essential for cardiac function and development [51]. Collectively, these data suggest that non-coding RNAs are a key component of the TNFα-mediated proinflammatory transcriptome in cardiomyocytes.

## Discussion

### Understanding the proinflammatory cardiomyocyte transcriptome using AC16 cells as a model

Heart disease remains the primary cause of mortality worldwide. Understanding the biology and function of cardiomyocytes is critical to discovering and reducing the causes of cardiac diseases and preventing the progression from heart injury to eventual heart failure. A global view of the proinflammatory cardiomyocyte transcriptome is a key component of our overall understanding. Our integrated genomic analyses using AC16 human cardiomyocytes as a model have helped to identify ~30,000 expressed transcripts under basal and proinflammatory stress conditions, including protein-coding, as well as a wide variety of non-coding, transcripts. Our analyses serve as a guide for studying functionally annotated as well as unannotated Pol I, II, and III transcripts, and our data are an excellent resource for understanding the cardiomyocyte transcriptomes and the transcriptomes of related cell types.

### Dynamic regulation of the AC16 transcriptome

A large fraction (18%) of the AC16 Pol II transcriptome is regulated by TNFα, which occurs extremely rapidly, with a large number of transcripts affected within 10 minutes of TNFα treatment (Figure 3). This regulation is highly dynamic, with the maximum regulation for most up- and downregulated transcripts occurring after 30 minutes of TNFα treatment, with a return to basal state at 120 minutes (Figure 4). Such a dynamic regulatory pattern is consistent with the oscillatory pattern of NF-κB nuclear translocation and gene activation [22] and is likely to play a key role in the signaling outcomes that drive cardiomyocyte biology in response to TNFα. Unlike a mitogenic growth response, which upregulates the expression of Pol I and III transcripts to meet the increased protein synthesis demands of proliferating cells [13,14], the proinflammatory response has little effect on the expression of Pol I and III transcripts (Figure 5). These differences reflect the different needs of cells responding to mitogenic and stress signals.

Strikingly, we observed that a large fraction (~80%) of TNFα-responsive transcription is downregulated following TNFα treatment (Figure 3C). In this regard, the loss of GRO-seq signal (i.e., active Pol II-mediated transcription) was accompanied by a loss of Pol II protein on the downregulated genes (Figure 4), as expected. Little is known about the mechanisms of gene repression during proinflammatory responses, especially at a global level. Potential mechanisms of repression include: (1) active repression associated with the recruitment of transcriptional corepressors to target genes, (2) release of transcriptional activators, or (3) passive redistribution of the Pol II transcription machinery to other highly induced genes. Interestingly, in general, NF-κB is recruited to TNFα-activated genes, but not TNFα-repressed genes in response to TNFα treatment (Figure 4C), suggesting that the gene repression we observed is not due to modulation of NF-κB binding. Other transcription factors may play a role in downregulation; for example, motif analyses of the downregulated gene promoters revealed a significant enrichment of transcription factor Sp1 binding sites (data not shown).

### A functional link between inflammation and cardiomyocyte function at the transcriptional level

The temporal regulation of gene expression in AC16 cells in response to TNFα, as reflected in our GRO-seq analyses, serves as a transcriptional readout of the sequential shift in cellular responses during the time course of treatment (Figure 7A). The upregulated and downregulated biological responses are closely related to cardiac function and indicate a shift from a basal cellular state to a proinflammatory stress-defense state. The transcripts driving these biological responses include those encoding proinflammatory mediators (e.g., *NFKB1, IL8)* and cell death-related factors (e.g., *TNF*, *CFLAR, APLF*), which are induced during the acute proinflammatory response. In addition, the expression of transcripts critical for maintaining normal cardiomyocyte function (e.g., *TCF21, CALM1*) is disrupted by TNFα treatment. Co-regulated transcripts may share a similar regulatory mechanism or be functionally related. For instance, we identified TNFα-regulated microRNA precursors, which are further processed into several critical cardiac-associated microRNAs (e.g., mir-21 and let-7 family members) that target mRNAs required for cardiac function [49-51].

## Conclusions

**Figure 9:**
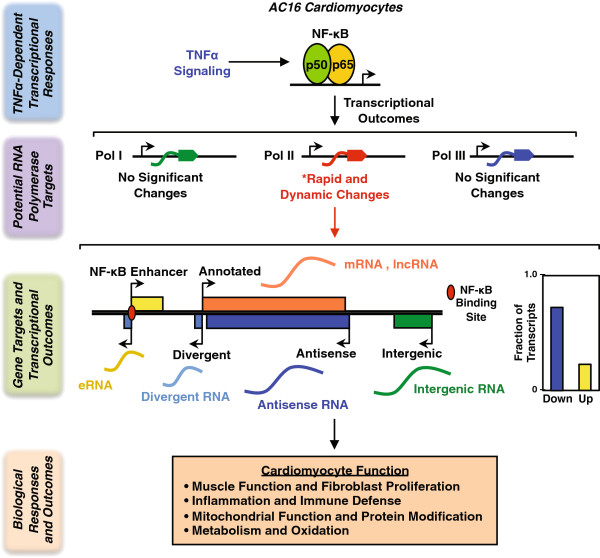
**TNFα signaling results in RNA polymerase II-dependent transcriptional outcomes and downstream biological effects in human cardiomyocytes.** TNFα signaling activates an NF-κB-mediated transcriptional program in AC16 cardiomyocytes. Integrated genomic analyses capture transcriptome alterations for all expressed Pol I, II, III transcripts in inflammatory cardiomyocytes. Pol II transcripts are the main drivers of the rapid and dynamic transcriptome changes, whereas non-Pol II transcripts show limited responses to TNFα . The Pol II dependent globally transcriptome changes include both protein coding and non-coding transcripts, which orchestrate the subsequent biological effects of inflammation on cardiomyocytes.

Collectively, our studies show how cells reorganize their transcriptomes to respond to proinflammatory signals, doing so in a manner that is distinctly different than response to other cellular signals (e.g., mitogenic; [13]). The dynamic transcriptome changes that we observed reflect the time-dependent shifting of biological processes in cardiomyocytes in response to TNFα (Figure 9). Moreover, our results suggest that proinflammatory stimulation is sufficient to capture many of the hallmarks of cardiovascular disease, suggesting a central role for the NF-κB pathway in heart disease.

## Methods

### Cell culture and treatments

AC16 human adult ventricular cardiomyocyte cells [52] were purchased from the American Type Cell Culture (ATCC). The cells were maintained in DMEM F-12 supplemented with 12.5% fetal bovine serum. TNFα was purchased from PeproTech (cat. no. 300-01A) and the IKKα/β inhibitor *BAY-*11-7082 was purchased from Calbiochem (cat. no. 196870). For TNFα treatments, the cells were grown to 75% confluence, switched to serum-free medium for 24 hours, and then treated with TNFα (25 ng/ml) for the indicated time. For experiments with BAY-11-7082, the cells were pretreated with the inhibitor (5 μM) or DMSO vehicle for 1 hour prior to treatment with TNFα for 30 minutes.

### Antibodies

The antibodies used were as follows: NF-κB p65 (Abcam; cat. no. ab7970), Pol II (Santa Cruz; cat. no. SC-899 and SC-900, mixed in a 1:4 ratio), β-tubulin (Abcam; cat. no.ab6046), SNRP70 (Abcam; cat. no ab51266), c-Fos (H-125, Santa Cruz; cat. no. sc-7202), c-Jun (H-79, Santa Cruz; cat. no. sc-1694), and NF-κB p50 (Abcam; cat. no. ab7971).

### Cell fractionation, extraction, and western blotting

For the cytoplasmic and nuclear extraction experiments shown in Figure 1A, AC16 cells were seeded at ~3 × 10^6^ cells per 15 cm diameter plate and treated as described above. After collecting the cells, extracts of the cytoplasmic and nuclear fractions were made according to the protocol provided with the Sigma CelLytic™ NuCLEAR™ Extraction Kit. Specifically, the cells were swollen in isotonic buffer [10 mM Tris–HCl, pH 7.5, 2 mM MgCl_2_, 3 mM CaCl_2_, 0.3 M sucrose, 1 mM DTT, and 1x Roche Complete Protease Inhibitor Cocktail (RCPIC)] on ice for 15 minutes and lysed by the addition of 0.6% IGEPAL CA-630 detergent with vortexing. The lysates were centrifuged and the supernatants were collected as the cytoplasmic fraction. The crude nuclear pellet was washed once with isotonic buffer, resuspended in extraction buffer (20 mM HEPES, pH 7.9, 1.5 mM MgCl_2_, 0.42 M NaCl, 0.2 mM EDTA, 25% v/v glycerol, 1 mM DTT, and 1× RCPIC), and vortexed vigorously for 20 minutes at 4°C. The resuspended nuclear material was then centrifuged and the supernatant taken as the nuclear extract. For each fraction under the indicated conditions, 40 μg protein was analyzed on an 8% polyacrylamide-SDS gel and transferred to a nitrocellulose membrane. Western blotting was performed with the appropriate primary and secondary antibodies.

For the whole cell extraction and Western blotting experiments shown in Figure 8, AC16 cells were seeded at ~1 × 10^6^ cells per 10 cm diameter plate and treated as described above. After collecting the cells, whole cell extracts were made in lysis buffer [50 mM Tris–HCl, pH 7.9, 150 mM NaCl, 1% NP-40, 0.5% Na deoxycholate (DOC), 1% SDS, 1 mM DTT, and 1x Roche Complete Protease Inhibitor Cocktail (RCPIC)] on ice for 30 minutes. The cell lysates were sonicated by using a Bioruptor UC200 at the high setting for a 5-minute cycles of 30 seconds on and 60 seconds off to release the chromatin bound proteins. The lysates were centrifuged and the supernatants were collected as the whole cell extracts. 20 μg protein was analyzed on an 12% polyacrylamide-SDS gel and transferred to a nitrocellulose membrane. Western blotting was performed with the appropriate primary and secondary antibodies.

### Bio-plex cytokine assay

AC16 cells were seeded at ~1 × 10^5^ cells per well in 6-well plates and treated as described above. The cell culture supernatants were collected and BSA was added as a carrier protein to a final concentration of 0.6%. The supernatants were centrifuged at 1,000 × g for 15 minutes at 4°C and 10,000 × g for 10 minutes at 4°C to remove cells and cell debris, respectively. The supernatants were assayed on a custom designed Bio-Plex Pro™ cytokine assay chip from Bio-rad to quantify the secretion of IL-6, IL-8 and MCP-1 according to the manufacturer’s instructions and run on a Bio-Plex 200 reader.

### RNA isolation and RT-qPCR

AC16 cells were seeded at ~1 × 10^5^ cells per well in 6-well plates and treated as described above. After collecting the cells, total RNA was isolated using TRIzol Reagent (Life Technologies) according to the manufacturer’s protocol. Total RNA was reverse transcribed using oligo (dT) primers and M-MLV reverse transcriptase, and was then subjected to real-time quantitative PCR (qPCR) using gene-specific primers:

• NFKB1_Fwd: 5′-CAGTGGTGCCTCACTGCTAA-3′

• NFKB1_Rev: 5′-GGACAACGCAGTGGAATTTT-3′

• IL6_Fwd: 5′-ATTCTGCGCAGCTTTAAGGA-3′

• IL6_Rev: 5′-GAGGTGCCCATGCTACATTT-3′

• TBP_Fwd: 5′-ATGTTGAGTTGCAGGGTGTG-3′

• TBP_Rev: 5′-CCCAGATAGCAGCACGGTAT-3′

All target gene expression was normalized to TBP expression. Each experiment was conducted with a minimum of three biological replicates.

### Chromatin immunoprecipitation-sequencing (ChIP-seq)

#### ChIP

ChIP was performed as described previously [53,54] with a few modifications [55]. AC16 cells were seeded at ~3 × 10^6^ cells per 15 cm diameter plate and treated as described above. The cells were cross-linked with 1% paraformaldehyde in PBS for 10 minutes at 37°C and quenched in 125 mM glycine in PBS for 5 minutes at 4°C. The cells were then collected and lysed in Farnham lysis buffer (5 mM PIPES pH 8.0, 85 mM KCl, 0.5% NP-40, 1 mM DTT, and 1x RCPIC). A crude nuclear pellet was collected by centrifugation, resuspended in lysis buffer (1% SDS, 10 mM EDTA, 50 mM Tris–HCl, pH 7.9, 1 mM DTT, and 1x RCPIC), and incubated on ice for 10 minutes. The chromatin was sheared at 4°C by sonication using a Bioruptor UC200 at the high setting for four 5-minute cycles of 30 seconds on and 60 seconds off to generate chromatin fragments of ~300 bp in length. The soluble chromatin was diluted 1:10 with dilution buffer (20 mM Tris–HCl, pH 7.9, 0.5% Triton X-100, 2 mM EDTA, 150 mM NaCl, 1 mM DTT and 1x RCPIC) and pre-cleared with protein A agarose beads. The pre-cleared supernatant was used in immunoprecipitation reactions with antibodies against the factor of interest or with rabbit IgG as a control. The immunoprecipitated material was washed once with low salt wash buffer (20 mM Tris–HCl, pH 7.9, 2 mM EDTA, 125 mM NaCl, 0.05% SDS, 1% Triton X-100, 1 μM aprotinin, and 1 μM leupeptin), once with high-salt wash buffer (20 mM Tris–HCl, pH 7.9, 2 mM EDTA, 500 mM NaCl, 0.05% SDS, 1% Triton X-100, 1 μM aprotinin, and 1 μM leupeptin), once with LiCl wash buffer (10 mM Tris–HCl, pH 7.9, 1 mM EDTA, 250 mM LiCl, 1% NP-40, 1% sodium deoxycholate, 1 μM aprotinin, and 1 μM leupeptin), and once with 1x Tris-EDTA (TE). The immunoprecipitated material was eluted in elution buffer (100 mM NaHCO_3_, 1% SDS) and was then digested with proteinase K and RNase H to remove protein and RNA, respectively. The immunoprecipitated genomic DNA was then extracted with phenol:chloroform:isoamyl alcohol and precipitated with ethanol.

#### ChIP-seq library preparation

The immunoprecipitated DNA was purified further using the MinElute PCR Purification Kit from Qiagen. After purification, 50 ng of ChIPed DNA for each condition was used to generate libraries for sequencing, as previously described [56], with some modifications. Briefly, the DNA was end-repaired and a single “A”-base overhang was added using the Klenow fragment of E. coli DNA polymerase. The A-modified DNA was ligated with Illumina sequencing adaptors using the Illumina TruSeq DNA Sample Prep Kit. The ligated DNA (250 ± 25 bp) was size-selected by agarose gel electrophoresis and extraction, amplified by PCR, and purified using AmPure beads (Beckman Coulter). The final libraries were subjected to QC (size, purity, adapter contamination) and sequenced using an Illumina Hiseq 2000 per the manufacturer’s instructions.

### ChIP-seq data analyses

NF-κB p65 and Pol II ChIP-Seq data in control and TNFα-treated AC16 cells were generated in the experiments described herein. In addition, existing datasets were downloaded from the NCBI’s GEO (Gene Expression Omnibus) database as listed below, and analyzed:

• GSM807734 HumanAdultHeart_acCBP-p300_ChIP-seq [57]

• GSM706848 Fetal_Heart.H3K4me1 [58]

• GSM733755 Bernstein_HSMM_H3K27ac [59]

• GSM1022657 UW_ChipSeq_HCM_CTCFRep1 [60]

• GSM1022677 UW_ChipSeq_HCM_CTCFRep2 [60]

• GSM935372 Harvard_ChipSeq_K562_RPC155_std [59]

• GSM935343 Harvard_ChipSeq_K562_TFIIIC-110_std [59]

The ChIP-seq reads were aligned to the hg19 human reference genome using the Bowtie software package [61]. Mapped reads were further converted to (1) “bed” files for later Metagene and read-density analyses and (2) “wiggle” files counting reads in non-overlapping 200-bp windows across the genome for presentation as genome browser tracks by using the BEDTools software package [62].

### Global run-on-sequencing (GRO-seq)

#### Isolation of nuclei

AC16 cells were seeded at ~3 × 10^6^ cells per 15 cm diameter plate and treated as described above. The cells were washed three times with ice-cold PBS, swollen osmotically, and collected in ice-cold lysis buffer [10 mM Tris–HCl, pH 7.4, 0.5% NP-40, 3 mM CaCl_2_, 2 mM MgCl_2_, 1 mM DTT, 1x RCPIC, and SUPERase•In™ (Ambion)] and centrifuged at 500 × g for 5 min at 4°C. The cells were then resuspended in 1.5 ml of lysis buffer and pipetted up and down through a narrow tip opening 20 times to release the nuclei. The nuclei were washed twice with a large volume of lysis buffer, and the washed nuclear pellets were resuspended in freezing buffer (50 mM Tris–HCl, pH 8.3, 40% glycerol, 5 mM MgCl_2_, 0.1 mM EDTA), counted, and stored in 100 μl aliquots containing 5 × 10^6^ nuclei.

#### GRO-seq library preparation

GRO-seq libraries were generated from two biological replicates of AC16 cells under the indicated treatment conditions, as previously described [18], but with limited modifications described previously [13]. The TNFα time course GRO-seq libraries were sequenced using an Illumina Genome Analyzer (GAIIx). For the α-amanitin experiments, the isolated nuclei were treated with 1 μg/ml α-amanitin (Sigma, cat. no. A2263) for 15 minutes on ice prior to the run-on reaction. The libraries generated from α-amanitin-treated nuclei were amplified with indexed primers containing barcodes according to the Illumina TrueSeq small-RNA library prep kit, then sequenced using an Illumina Hiseq 2000.

### GRO-seq data analyses

GRO-seq data were processed and mapped using a computational pipeline described previously [13], with limited modifications. Briefly, all reads longer than 32 bp were aligned to the hg19 human reference genome (including autosomes, X chromosome, and a complete copy of rDNA repeats) using the SOAP2.21 software package [63].

#### Transcript calling

Unbiased transcript calling was performed using an algorithm based on a two-state hidden Markov model as described previously [13]. A shape parameter value of 5 was used for the non-transcribed-state emission probability and a value of 200 was used as the negative log of the transition probability from the transcribed state to the non-transcribed state. To map the relatively smaller non-Pol II transcripts more accurately in the α-amanitin GRO-seq datasets, values of 5 and 10, respectively, were used. In order to capture non-Pol II transcripts more effectively, the control signal was subtracted from the α-amanitin signal using a running maximum of window-size three (25 bp) and adding the baseline of the mean positive signal of the control. Transcripts were then called using an algorithm based on a two-state hidden Markov model as described above.

#### Functional definitions of called transcripts

Called transcripts were assigned to one of the following eight functional classes, according to the rules enumerated below. For all annotations, the following sources were used: RefSeq, GENCODE release 11, ENSEMBLE, lincRNAsTranscripts, repeat masker tracks (obtained using the UCSC genome browser; [64]), and mirBase 18 [64,65].

(1) *** Protein-coding transcript.*** A transcript with more than 20% of its sequence overlapping any well annotated protein-coding gene.

(2) *** Non-coding transcript.*** A transcript overlapping an annotated non-coding RNA gene, such as those encoding a miRNA, tRNA, snRNA, or lncRNA, without any restrictions on the size of the transcript or the quality of the overlap. By the standard definition, lncRNAs are non-protein-coding transcripts equal to or longer than 200 nucleotides in the mature (processed) form, whereas short ncRNAs are non-protein-coding transcripts shorter than 200 nucleotides in the mature (processed) form.

(3) *** Intergenic transcript.*** A transcript that does not overlap with an annotated gene. Examples are likely to include: (i) novel unannotated protein-coding and non-coding genes, (ii) enhancer transcripts, or (iii) post poly (A) transcription for some well-annotated Pol II genes with low expression levels.

(4) ** Enhancer transcripts (eRNAs).** A pair of short (< 9 kb) bidirectionally transcribed intergenic transcripts that do not significantly overlap annotated transcripts [20]. We call those that overlap an NF-κB binding site (i.e., ±1000 kb from the center of an NF-κB p65 peak as defined by ChIP-seq) “NF-κB binding site eRNAs” and those that do not overlap an NF-κB binding site “non-NF-κB binding site eRNAs”. Putative target genes for the identified enhancers marked by the eRNAs were defined by searching for the nearest protein-coding or lncRNA gene in either direction.

(5) *** Divergent transcript.*** A transcript that overlaps the 5′ promoter driving expression of a detected primary transcript, such as an mRNA or a lncRNA. A divergent transcript was only included if (1) >10% of the transcript overlapped the proximal region of a promoter (± 500 bp relative to the TSS) driving expression of a primary transcript >1 kb in size on the opposite strand and (2) the transcript was <50% the size of the primary transcript, which effectively excluded divergent enhancer-transcript pairs.

(6) *** Antisense transcript.*** A transcript that runs antisense to a protein-coding gene or lncRNA gene and has >20% of its sequence overlapping >20% of an annotated protein-coding gene or lncRNA gene on the opposite strand.

(7) *** Repeat transcript.*** A transcript with more than 50% of its sequence overlapping genomic regions identified in the RepeatMasker track in the UCSC Genome Browser.

(8) *** Other genic transcript.*** A transcript that has a poor match to existing annotations, but cannot be unambiguously classified as “unannotated” or “intergenic”. Transcripts in this category overlap any segment of a gene annotation on either strand, but shows <20% matching to the annotation. Examples in this category may include: (1) genes with promoter proximal RNA Pol II pausing, but very low levels of transcription in the gene body, (2) divergent transcripts from internal start sites (antisense), (3) intronic enhancer transcripts, or (4) short cryptic transcripts of unknown function.

#### Determining regulation by TNFα

Regulation in response to TNFα treatment was determined using the edgeR software package [21] with a false discovery rate (FDR)-corrected q value threshold of < 0.001, as described previously [13].

### Other genomic data analyses

#### Metagene analyses

Metagene analyses were performed to illustrate the distribution of average GRO-seq and ChIP-seq read densities ±5 kb surrounding fixed genomic landmarks (e.g., TSSs, the midpoint of paired eRNAs, center of ChIP-seq peaks) using the metagene functions in our GRO-seq package, as previously described [13,20,66].

#### Motif-finding analyses

De novo motif analyses for a 1 kb region around the center of the overlap of paired eRNAs were performed using MEME [33] with a “–zoops” setting (zero or one occurrence per sequence) and a motif size between 8 and 15. The outputs of MEME were matched to known motifs using STAMP [34] with default settings.

#### Gene ontology analyses

Gene ontology (GO) analyses were performed using the Genomic Regions Enrichment of Annotations Tool (GREAT), version 2.0.2 [47], with the following association rule: Basal + extension: 5000 bp upstream, 1000 bp downstream, 1,000,000 bp max extension, curated regulatory domains included.

#### Gene set enrichment analyses

Gene Set Enrichment Analysis (GSEA), version 2.0.12 [67], was used to identify all enriched GO terms at each TNFα treatment time point using the 0 minute condition as a control with a set of GO terms from humans (http://download.baderlab.org/EM_Genesets/September_02_2011/Human/symbol/GO/Human_GO_bp_no_GO_iea_symbol.gmt). For ranked inputs to GSEA, we used pre-ranked gene lists based on edgeR differential analysis after filtering out gene sets whose size was greater than 500 or less than 25. Specifically, significantly regulated genes (FDR < 0.1%) were placed at the top or bottom of the list and ordered by descending or ascending fold changes, respectively. Less significantly regulated genes (FDR ≥ 0.1%) were placed in the middle of the list and ordered by ascending or descending p-values.

#### Hierarchical clustering and heatmaps

Hierarchical clustering was performed using the results of the GSEA. GO terms with the top ten Normalized Enrichment Scores (NESs) were selected and combined from both the upregulated and downregulated GO terms in each time point, compared to the 0 min treatment condition. Heatmaps were generated using the heatmap.2 function in the gplots package in R with default parameters. Inputs for the heatmaps included (1) normalized GRO-seq signals (median-centered and scaled relative to the 0 min time point for expression analyses; e.g. Figure 3B) and (2) NESs (e.g., Figure 7A). For the latter, heatmaps comparing later to earlier time points (e.g., 10 min vs. 30 min, or 30 min vs. 120 min) were generated in a similar manner, but using different edgeR outputs for the comparisons.

### Data access

The GRO-seq and ChIP-seq data sets described herein are available from the NCBI Gene Expression Omnibus (GEO) (http://www.ncbi.nlm.nih.gov/geo/) using accession number GSE51225.

## Competing interests

The authors declare that they have no competing interests.

## Authors’ contributions

RK and XL established and developed the AC16 cardiomyocyte proinflammatory response system. RK performed the initial gene-specific and genomic experiments that influenced the design and direction of the current study. XL and WLK conceived and designed the experiments. XL performed all of the wet lab experiments, generated the GRO-seq and ChIP-seq data sets, analyzed the ChIP-seq data, and performed the gene ontology analyses. CGD developed the GRO-seq data analysis pipeline and performed the initial GRO-seq data analyses. MC performed the final GRO-seq data analyses, as well as the gene set enrichment and motif analyses. XL and WLK analyzed and integrated the data, and assembled the data into Figures. XL wrote the initial draft of the paper, which was edited by WLK and the other authors. All authors read and approved the final manuscript.

## Supplementary Material

Additional file 1 **GRO-seq identifies non-coding transcripts relevant to cardiac biology whose expression is regulated by TNFα *[Related to Figure*** 2 ***].*** Genome browser track representations of GRO-seq read density distributions for different TNFα-regulated cardiac-related transcripts. Scale bars and annotations are shown. The DNA strands are indicated. **(A) ***CFLAR* and *CFLAR-AS; ***(B) ***MALAT1; ***(C) ***mir-21* precursor (*MIR21*); **(D) ***MIRLET7BHG.* Click here for file

Additional file 2 **Enhancer transcription is inhibited by α-amanitin *[Related to Figure*** 5 ***].*** Nuclei isolated from AC16 cells were incubated on ice with α-amanitin for 15 min. prior to the run-on reaction and were then subjected to GRO-seq analysis. The plots are metagene representations of the average GRO-seq read distributions ± 4 kb around the midpoint of overlap of bidirectionally transcribed eRNAs. Click here for file

Additional file 3 **Genome browser views of GRO-seq and ChIP-seq data for non-Pol II genes *[Related to Figure*** 5 ***].*** Non-Pol II transcription units in AC16 cells were identified by GRO-seq using α-amanitin. The top panel in each set shows genome browser tracks of GRO-seq data under control and α-amanitin-treated conditions, or with TNFα treatment for 30 minutes. The bottom panel in each set shows genome browser tracks of ChIP-seq data for RPC155 in K562 cells, CTCF in HCM cells, and Pol II in AC16 cells with and without TNFα treatment. **A)** a tRNA transcription unit on Chr1 (tRNA2-GlyCCC). **B)** a non-Pol II transcription unit located in the first intron of the protein-coding gene *PMF1*. **C)** three intergenic non-Pol II transcription units on Chr5 (140,084,426 –140,112,361). Click here for file

Additional file 4 **NF-κB-dependent enhancers identified by GRO-seq and motif analyses are enriched for NF-κB binding *[Related to Figure*** 6 ***].*** GRO-seq was used to identify 208 NF-κB enhancers in AC16 cells, which are enriched in NF-κB motifs (see Figure 6D). As shown in the graph, they are also enriched for NF-κB p65 binding + TNFα relative to non-NF-κB enhancers, as determined by ChIP-seq. The graph is a metagene representation of the average ChIP-seq read distributions for NF-κB p65 shown relative to the midpoint of overlap of the bidirectionally transcribed eRNAs (± 4 kb) for NF-κB enhancers and non-NF-κB enhancers ± TNFα. Click here for file

Additional file 5 **Enriched GO terms in gene set enrichment analyses of TNFα-regulated protein-coding genes *[Related to Figure*** 7 ***].*** To relate transcriptome changes to biological processes in AC16 cells, we performed gene ontology and gene set enrichment analyses on TNFα up- and downregulated protein-coding gene sets identified by GRO-seq. Click here for file

Additional file 6 **Gene ontology analyses of different classes of transcripts *[Related to Figure*** 7 ***].*** Gene ontology analyses of different classes of transcripts. Due to incomplete availability of annotations or limited direct functional information, assignment of GO terms to non-coding transcript can be difficult. We performed gene ontology analyses using the Genomic Regions Enrichment of Annotations Tool (GREAT), which aids in the predicting the molecular function, associated biological processes, and disease associations based on the genomic region of interest and nearby genomic regions [1]. The table below lists the top enriched GO biological processes for **(A)** TNFα upregulated protein-coding genes and adjacent genomic regions, **(B)** TNFα downregulated protein-coding genes and adjacent genomic regions, **(C)** TNFα upregulated lncRNA genes and adjacent genomic regions, **(D)** TNFα upregulated enhancers and adjacent genomic regions, and **(E)** TNFα upregulated antisense RNA genes and adjacent genomic regions. **Binom FDR Q-Val** = Binomial false discovery rate Q-value (adjusted p-value found using an optimized FDR approach; the minimum FDR at which the test is called significant.). **Binom Fold Enrichment** = Binomial fold enrichment (observed/expected). Click here for file
