# Supplementary figures and images for: Dynamic reorganization of the AC16 cardiomyocyte transcriptome in response to TNFα signaling revealed by integrated genomic analyses
Source: BMC Genomics. 2014 Feb 24;15:155. doi: 10.1186/1471-2164-15-155 (PMC3945043; doi:10.1186/1471-2164-15-155)

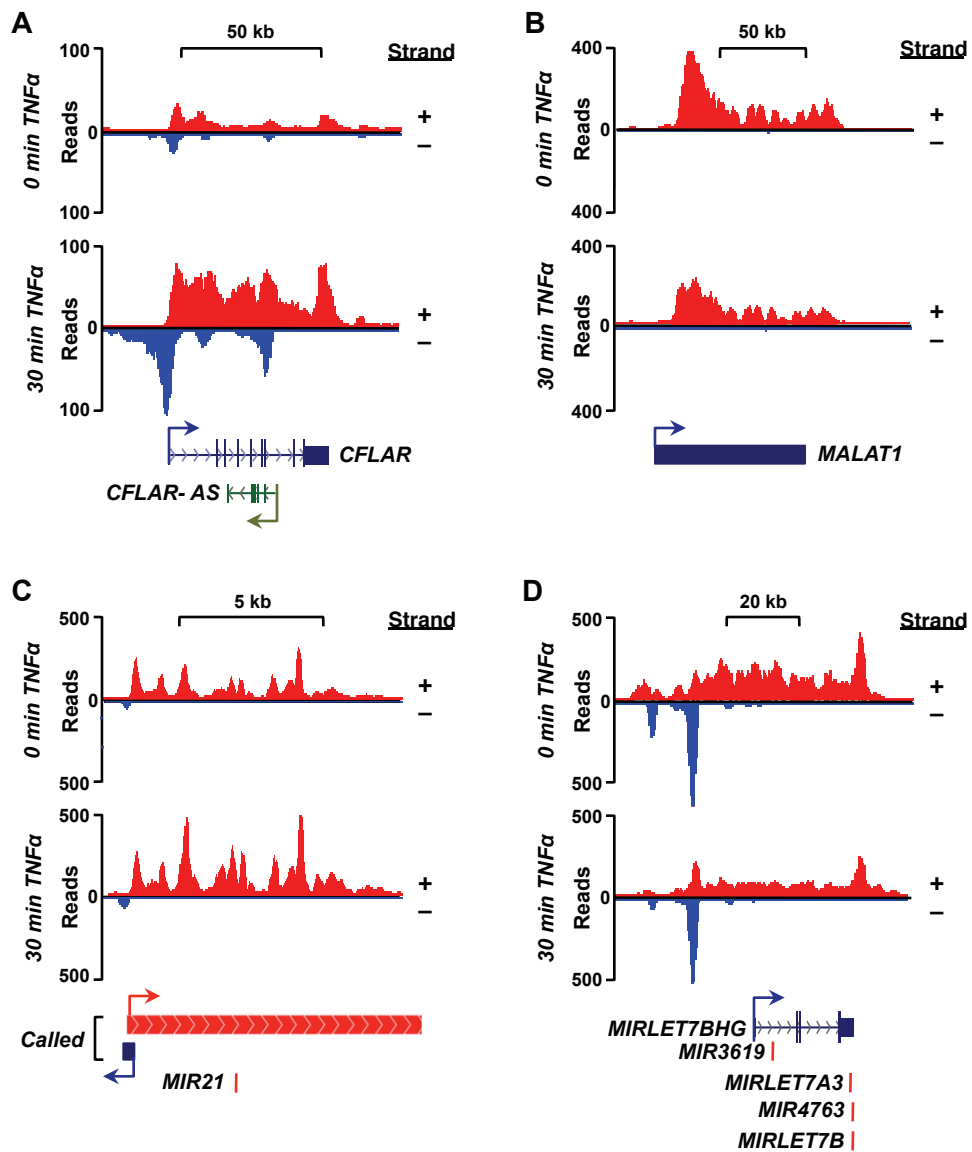

Supplement: Additional file 1 — GRO-seq identifies non-coding transcripts relevant to cardiac biology whose expression is regulated by TNFα [Related to Figure 2 ]. Genome browser track representations of GRO-seq read density distributions for different TNFα-regulated cardiac-related transcripts. Scale bars and annotations are shown. The DNA strands are indicated. (A) CFLAR and CFLAR-AS; (B) MALAT1; (C) mir-21 precursor (MIR21); (D) MIRLET7BHG. [file 1471-2164-15-155-S1.pdf]

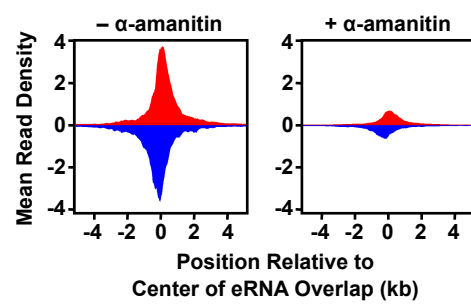

Supplement: Additional file 2 — Enhancer transcription is inhibited by α-amanitin [Related to Figure 5 ]. Nuclei isolated from AC16 cells were incubated on ice with α-amanitin for 15 min. prior to the run-on reaction and were then subjected to GRO-seq analysis. The plots are metagene representations of the average GRO-seq read distributions ± 4 kb around the midpoint of overlap of bidirectionally transcribed eRNAs. [file 1471-2164-15-155-S2.pdf]

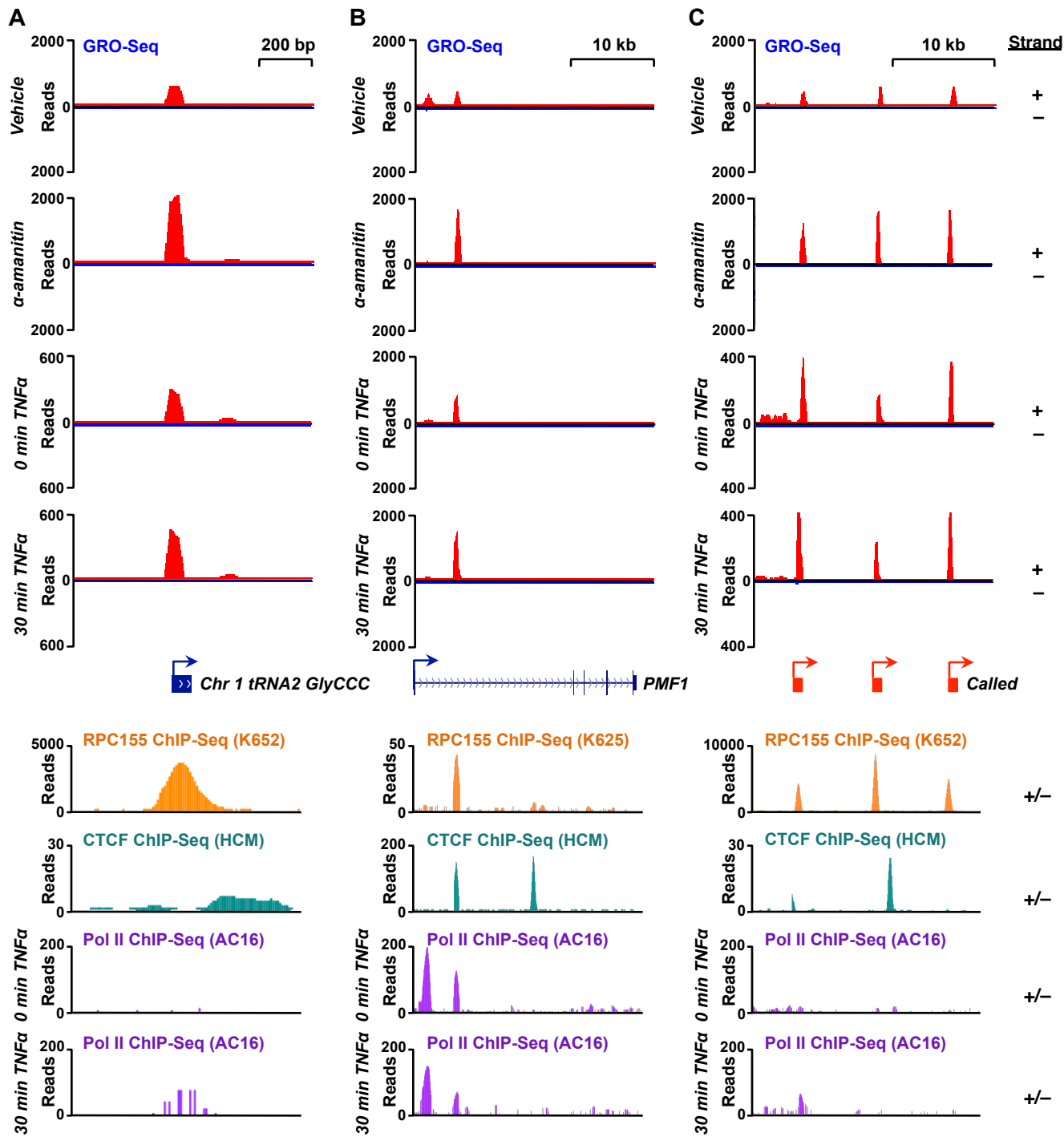

Supplement: Additional file 3 — Genome browser views of GRO-seq and ChIP-seq data for non-Pol II genes [Related to Figure 5 ]. Non-Pol II transcription units in AC16 cells were identified by GRO-seq using α-amanitin. The top panel in each set shows genome browser tracks of GRO-seq data under control and α-amanitin-treated conditions, or with TNFα treatment for 30 minutes. The bottom panel in each set shows genome browser tracks of ChIP-seq data for RPC155 in K562 cells, CTCF in HCM cells, and Pol II in AC16 cells with and without TNFα treatment. A) a tRNA transcription unit on Chr1 (tRNA2-GlyCCC). B) a non-Pol II transcription unit located in the first intron of the protein-coding gene PMF1. C) three intergenic non-Pol II transcription units on Chr5 (140,084,426 –140,112,361). [file 1471-2164-15-155-S3.pdf]
