# Supplementary material for: Dynamic reorganization of the AC16 cardiomyocyte transcriptome in response to TNFα signaling revealed by integrated genomic analyses
Source: BMC Genomics. 2014 Feb 24;15:155. doi: 10.1186/1471-2164-15-155 (PMC3945043; doi:10.1186/1471-2164-15-155)
Supplement: Additional file 4 — NF-κB-dependent enhancers identified by GRO-seq and motif analyses are enriched for NF-κB binding [Related to Figure 6 ]. GRO-seq was used to identify 208 NF-κB enhancers in AC16 cells, which are enriched in NF-κB motifs (see Figure 6D). As shown in the graph, they are also enriched for NF-κB p65 binding + TNFα relative to non-NF-κB enhancers, as determined by ChIP-seq. The graph is a metagene representation of the average ChIP-seq read distributions for NF-κB p65 shown relative to the midpoint of overlap of the bidirectionally transcribed eRNAs (± 4 kb) for NF-κB enhancers and non-NF-κB enhancers ± TNFα. [file 1471-2164-15-155-S4.pdf]

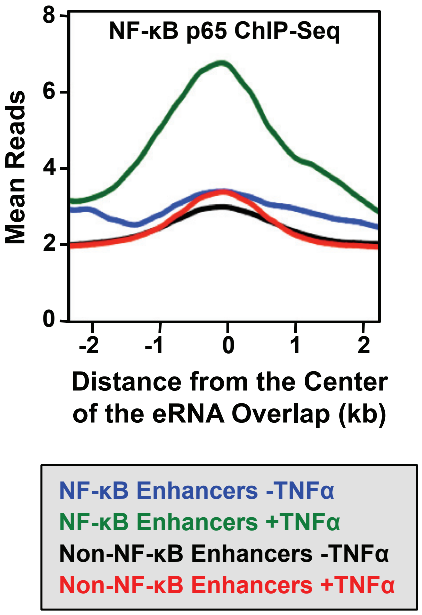

NF-κB p65 ChIP-Seq
Mean Reads
8
6
4
2
0
-2
-1
0
1
2
Distance from the Center
of the eRNA Overlap (kb)
NF-κB Enhancers -TNFα
NF-κB Enhancers +TNFα
Non-NF-κB Enhancers -TNFα
Non-NF-κB Enhancers +TNFα
