# Supplementary material for: Dynamic reorganization of the AC16 cardiomyocyte transcriptome in response to TNFα signaling revealed by integrated genomic analyses
Source: BMC Genomics. 2014 Feb 24;15:155. doi: 10.1186/1471-2164-15-155 (PMC3945043; doi:10.1186/1471-2164-15-155)
Supplement: Additional file 5 — Enriched GO terms in gene set enrichment analyses of TNFα-regulated protein-coding genes [Related to Figure 7 ]. To relate transcriptome changes to biological processes in AC16 cells, we performed gene ontology and gene set enrichment analyses on TNFα up- and downregulated protein-coding gene sets identified by GRO-seq. [file 1471-2164-15-155-S5.pdf]

A. Cluster I: Muscle function and fibroblast proliferation	
**GO Terms**	**ID**
muscle filament sliding	GO:0030049
actin-myosin filament sliding	GO:0033275
actin-mediated cell contraction	GO:0070252
positive regulation of fibroblast proliferation	GO:0048146
epithelial to mesenchymal transition	GO:0001837

B. Cluster II: Inflammation and immune responses	
**GO Terms**	**ID**
inflammatory response	GO:0006954
regulation of type I interferon-mediated signaling pathway	GO:0060338
regulation of cytokine-mediated signaling pathway	GO:0001959
regulation of production of molecular mediator of immune response	GO:0002700
regulation of cell adhesion mediated by integrin	GO:0033628
cytokine receptor binding	GO:0005126
positive regulation of inflammatory response	GO:0050729
cytokine activity	GO:0005125
cellular response to interferon-gamma	GO:0071346
response to interferon-gamma	GO:0034341
interferon-gamma-mediated signaling pathway	GO:0060333

C. Cluster III: Translational regulation and viral expression	
**GO Terms**	**ID**
translational termination	GO:0006415
translational elongation	GO:0006414
protein complex disassembly	GO:0043241
viral transcription	GO:0019083
viral genome expression	GO:0019080
cytosolic ribosome	GO:0022626
translational initiation	GO:0006413
SRP-dependent cotranslational protein targeting to membrane	GO:0006614
establishment of protein localization to endoplasmic reticulum	GO:0072599
cotranslational protein targeting to membrane	GO:0006613

D. Cluster IV: Mitochondrial function and protein modification	
**GO Terms**	**ID**
proton-transporting two-sector ATPase complex	GO:0016469
mitochondrial membrane part	GO:0044455
histone lysine methylation	GO:0034968
mitochondrial matrix	GO:0005759
protein methyltransferase activity	GO:0008276
protein-lysine N-methyltransferase activity	GO:0016279
lysine N-methyltransferase activity	GO:0016278
cellular respiration	GO:0045333

respiratory chain	GO:0070469
mitochondrial respiratory chain	GO:0005746
porphyrin-containing compound metabolic process	GO:0006778
tetrapyrrole metabolic process	GO:0033013

E. Cluster V: Metabolism and oxidation	
**GO Terms**	**ID**
oxidoreductase activity, acting on the CH-CH group of donors	GO:0016627
glutathione metabolic process	GO:0006749
peroxisomal matrix	GO:0005782
microbody lumen	GO:0031907
small molecule catabolic process	GO:0044282
organic acid catabolic process	GO:0016054
carboxylic acid catabolic process	GO:0046395
monocarboxylic acid catabolic process	GO:0072329
fatty acid beta-oxidation	GO:0006635
fatty acid catabolic process	GO:0009062
lipid oxidation	GO:0034440
fatty acid oxidation	GO:0019395
