# Supplementary material for: Dynamic reorganization of the AC16 cardiomyocyte transcriptome in response to TNFα signaling revealed by integrated genomic analyses
Source: BMC Genomics. 2014 Feb 24;15:155. doi: 10.1186/1471-2164-15-155 (PMC3945043; doi:10.1186/1471-2164-15-155)
Supplement: Additional file 6 — Gene ontology analyses of different classes of transcripts [Related to Figure 7 ]. Gene ontology analyses of different classes of transcripts. Due to incomplete availability of annotations or limited direct functional information, assignment of GO terms to non-coding transcript can be difficult. We performed gene ontology analyses using the Genomic Regions Enrichment of Annotations Tool (GREAT), which aids in the predicting the molecular function, associated biological processes, and disease associations based on the genomic region of interest and nearby genomic regions [1]. The table below lists the top enriched GO biological processes for (A) TNFα upregulated protein-coding genes and adjacent genomic regions, (B) TNFα downregulated protein-coding genes and adjacent genomic regions, (C) TNFα upregulated lncRNA genes and adjacent genomic regions, (D) TNFα upregulated enhancers and adjacent genomic regions, and (E) TNFα upregulated antisense RNA genes and adjacent genomic regions. Binom FDR Q-Val = Binomial false discovery rate Q-value (adjusted p-value found using an optimized FDR approach; the minimum FDR at which the test is called significant.). Binom Fold Enrichment = Binomial fold enrichment (observed/expected). [file 1471-2164-15-155-S6.pdf]

A. TNFα upregulated protein-coding genes and adjacent genomic regions		
**GO Biological Process**	**Binom FDR Q-Val**	**Binom Fold Enrichment**
defense response	6.89E-27	3.53
immune response	6.62E-24	3.54
response to stress	5.57E-22	2.02
immune system process	1.32E-20	2.48
inflammatory response	1.15E-15	4.29
response to wounding	3.20E-15	2.49
regulation of programmed cell death	4.82E-13	2.19
response to cytokine stimulus	1.09E-12	3.54
innate immune response	1.03E-12	3.64
regulation of cell death	1.08E-12	2.13
regulation of apoptosis	1.09E-12	2.17
regulation of cytokine production	2.89E-12	3.83
regulation of response to stress	3.44E-11	2.65
regulation of immune system process	2.86E-10	2.36
cellular response to cytokine stimulus	5.25E-10	3.89
positive regulation of response to stimulus	1.01E-09	2.10
cytokine-mediated signaling pathway	1.90E-09	4.44
multi-organism process	3.03E-09	2.21
positive regulation of cellular component movement	5.61E-09	3.29
response to other organism	6.27E-09	2.85

B. TNFα downregulated protein-coding genes and adjacent genomic regions		
**GO Biological Process**	**Binom FDR Q-Val**	**Binom Fold Enrichment**
cofactor metabolic process	2.00E-10	2.37
cofactor biosynthetic process	2.35972E-06	2.55
coenzyme metabolic process	5.39966E-05	2.01

C. TNFα upregulated lncRNA genes and adjacent genomic regions		
**GO Biological Processes**	**Binom FDR Q-Val**	**Binom Fold Enrichment**
regulation of cell proliferation	1.40E-07	2.94
multi-organism process	7.68E-08	3.64
response to mechanical stimulus	6.38E-07	8.79
regulation of apoptosis	2.40742E-06	2.86
response to stress	2.23523E-06	2.18
regulation of cell death	2.02791E-06	2.78
negative regulation of cellular process	1.99749E-06	2.02
regulation of programmed cell death	1.89378E-06	2.83
vasculature development	3.23846E-06	3.88
positive regulation of macromolecule metabolic process	3.14824E-06	2.38
response to lipopolysaccharide	3.52322E-06	6.90

response to wounding	3.59794E-06	3.05
blood vessel development	3.85106E-06	3.93
positive regulation of metabolic process	5.30154E-06	2.28
response to molecule of bacterial origin	4.31218E-06	6.44
regulation of immune system process	4.69096E-06	3.28
positive regulation of cell proliferation	9.04782E-06	3.25
response to peptidoglycan	9.17262E-06	32.4
regulation of response to stress	1.08144E-05	3.55
response to cytokine stimulus	1.27001E-05	4.66

D. TNFα upregulated enhancers and adjacent genomic regions		
**GO Biological Process**	**Binom FDR Q-Val**	**Binom Fold Enrichment**
regulation of response to stress	1.47E-08	3.19
multi-organism process	2.91E-08	2.71
regulation of cell proliferation	1.72E-07	2.20
regulation of cellular response to stress	7.31E-07	4.18
response to abiotic stimulus	1.09E-06	2.85
regulation of cytokine-mediated signaling pathway	2.03E-06	7.67
regulation of response to cytokine stimulus	1.89E-06	7.65
regulation of cytokine production	4.87E-06	3.83
negative regulation of transport	1.12E-05	3.79
cytokine-mediated signaling pathway	1.29E-05	4.87
regulation of myeloid cell differentiation	1.46E-05	5.43
positive regulation of cytokine production	1.43E-05	5.09
regulation of type I interferon-mediated signaling pathway	1.41E-05	17.5
regulation of immune system process	1.38E-05	2.42
cellular response to cytokine stimulus	1.66E-05	4.06
JAK-STAT cascade	2.05E-05	12.8
cell proliferation	2.46E-05	2.53
response to other organism	2.61E-05	3.05
response to cytokine stimulus	4.25E-05	3.21
cellular response to stress	5.81E-05	2.27

E. TNFα upregulated antisense RNA genes and adjacent genomic regions		
**GO Biological Process**	**Binom FDR Q-Val**	**Binom Fold Enrichment**
regulation of response to stress	1.47E-08	3.20
multi-organism process	2.91E-08	2.71
regulation of cell proliferation	1.72E-07	2.20
regulation of cellular response to stress	7.31E-07	4.18
response to abiotic stimulus	1.09E-06	2.85
regulation of cytokine-mediated signaling pathway	2.03E-06	7.67
regulation of response to cytokine stimulus	1.89E-06	7.65
regulation of cytokine production	4.87E-06	3.83

negative regulation of transport	1.12E-05	3.79
cytokine-mediated signaling pathway	1.29E-05	4.87
regulation of myeloid cell differentiation	1.46E-05	5.43
positive regulation of cytokine production	1.43E-05	5.09
regulation of type I interferon-mediated signaling pathway	1.41E-05	17.5
regulation of immune system process	1.38E-05	2.42
cellular response to cytokine stimulus	1.66E-05	4.06
JAK-STAT cascade	2.05E-05	12.8
cell proliferation	2.46E-05	2.53
response to other organism	2.61E-05	3.05
response to cytokine stimulus	4.25E-05	3.21
cellular response to stress	5.81E-05	2.27
